# Progression of visual cognition and neuropsychiatric symptoms in Huntington’s disease: a 1-year follow-up study across preclinical and clinical phases

**DOI:** 10.3389/fpsyg.2025.1609403

**Published:** 2025-10-06

**Authors:** Rocio Del Pino, Maria Ángeles Acera, Ane Murueta-Goyena, Beatriz Tijero, Marta Ruiz-Lopez, Johanne Somme, Silvia Pérez-Fernández, Javier Ruiz, Andrea Gabilondo, Rosario Sánchez-Pernaute, Iñigo Gabilondo, Tamara Fernández-Valle, Juan Carlos Gómez Esteban

**Affiliations:** ^1^Neurodegenerative Diseases Group, Biobizkaia Health Research Institute, Barakaldo, Spain; ^2^IKERBASQUE, The Basque Foundation for Science, Bilbao, Spain; ^3^Department of Neuroscience, University of the Basque Country (Universidad del Pais Vasco/Euskal Herriko Unibertsitatea), Leioa, Spain; ^4^Department of Neurology, Osakidetza, Cruces University Hospital, Barakaldo, Spain; ^5^Department of Neurology, Araba University Hospital, Vitoria-Gasteiz, Spain; ^6^Bioinformatics, Biostatistics and Information Systems Platform, Biobizkaia Health Research Institute, Barakaldo, Spain; ^7^Department of Neurology, Donostia University Hospital, San Sebastián, Gipuzkoa, Spain; ^8^Outpatient Mental Health Network of Gipuzkoa, San Sebastián, Spain

**Keywords:** cognition impairment, Huntington’s disease, neuropsychiatric symptoms, prodromal symptoms, visual cognition

## Abstract

**Introduction:**

Huntington’s disease (HD) is a progressive and complex neurodegenerative disorder marked by motor, psychiatric, and cognitive impairments. This study evaluates the progression of visual cognition across the HD spectrum—including pre-manifest with reduced penetrance (RP) alleles—as a potential early marker of disease progression. Secondary objectives assess changes in motor function, general cognition, and neuropsychiatric symptoms, and identify predictors of clinical trajectories. Exploratory analyses focused on the characterization of pre-manifest and RP individuals compared to HD-manifest carriers.

**Methods:**

We assessed 181 participants at baseline and 1-year follow-up: 40 pre-manifest HD, 30 early-manifest HD, 27 manifest HD, 6 RP, and 78 healthy controls (HC). Visual cognition, motor and general cognitive function, neuropsychiatric symptoms, premorbid intelligence, and quality of life was evaluated. Linear mixed models were applied, including models excluding HC and integrating years to estimated motor onset.

**Results:**

Visual cognition, especially visual memory and attention, emerged as a sensitive domain for early decline across the HD spectrum. Pre-manifest individuals showed significant worsening in visual memory compared to HC, while RP carriers exhibited additional changes in visual attention and processing speed despite preserved motor status. Excluding HC and using pre-manifest group as reference, significant longitudinal decline was confirmed in visual domains (memory, attention, processing speed, visuospatial abilities), and verbal fluency, with years to estimated onset and premorbid intelligence emerging as consistent predictors. Motor performance and general cognition declined mainly in early- and manifest carriers. Neuropsychiatric symptoms worsened across the spectrum, with manifest patients most affected. RP carriers presented the highest levels of suicidal ideation, significantly higher than pre-manifest individuals. Protective factors included higher premorbid intelligence, better daily functioning, quality of life, and lower baseline apathy and suicidal ideation.

**Conclusion:**

Visual cognitive decline— particularly memory and attention—represents an early and sensitive marker of disease progression in HD, even in asymptomatic carriers. Emotional vulnerability, including elevated suicidal ideation in RP individuals, further highlights the need for close monitoring of this under-characterized subgroup. These findings support the integration of visual cognition and neuropsychiatric assessments into early detection frameworks, with direct implications for personalized interventions and preventive strategies in prodromal stages.

## 1 Introduction

Huntington’s disease (HD) is a genetic, autosomal dominant neurodegenerative disorder characterized by complex and heterogeneous clinical presentation. While traditionally described across three major domains—progressive motor dysfunction, cognitive decline, and neuropsychiatric disturbances—its manifestations are highly variable and often extend beyond this framework, severely impacting patients’ autonomy and quality of life (QoL) (Hubers et al., 2013; Ross et al., 2014). The condition is caused by an unstable cytosine-adenine-guanine (CAG) triplet expansion in the HTT gene on chromosome 4 (Hoogeveen et al., 1993). Neuropathologically, HD involves extensive degeneration of the striatum (particularly the caudate and putamen) and cortical regions, but also affects other subcortical structures such as the globus pallidus, thalamus, and hypothalamus (Estrada-Sánchez and Rebec, 2013; Martínez and Rábano, 2002; Waldvogel et al., 2012). Disruption of the corticostriatal-cortical circuitry, which is crucial for motor and cognitive regulation, is a hallmark feature of the disease [4]. Understanding these early alterations is essential for timely diagnosis and intervention (Ross et al., 2014).

Although clinical diagnosis of HD primarily relies on the emergence of overt motor signs, increasing evidence suggests that non-motor symptoms, including cognitive and psychiatric changes, often precede motor onset by more than a decade (Halpin, 2020; Hicks et al., 2008; Murueta-Goyena et al., 2023). Early motor signs include chorea, bradykinesia, impaired balance and coordination, and oculomotor dysfunction—the latter often among the earliest detectable abnormalities (McAllister et al., 2021; Pino et al., 2025). Notably, cognitive deficits can manifest during the prodromal stage, correlating with early striatal atrophy detectable up to 10–15 years before diagnosis (Coppen et al., 2018; Duff et al., 2007; Verny et al., 2007). These cognitive changes include impairments in executive functions, working memory, processing speed, attention, and visuospatial skills, all of which compromise daily functioning and decision-making capacity (Labuschagne et al., 2016; Paulsen and Long, 2014; Radulovich Muñoz et al., 2019; Verny et al., 2007).

Visual cognition, a multidimensional construct involving visual attention, visuospatial abilities, visual memory, and perceptual processing, has emerged as an early and sensitive domain affected in HD (Eddy et al., 2016; Labuschagne et al., 2016; Pino et al., 2025; Radulovich Muñoz et al., 2019). Despite its clinical relevance, visual cognition has often been overlooked in longitudinal studies and remains poorly integrated into early diagnostic models. Recent studies have shown that impairments in visual memory, visuospatial abilities, and visuomotor integration can be detected in pre-manifest individuals and may distinguish them from controls with high sensitivity (Eddy et al., 2016; Labuschagne et al., 2016; Pino et al., 2025). Moreover, retinal changes assessed through optical coherence tomography (OCT) have been proposed as potential biomarkers of cognitive status in manifest HD, reinforcing the link between visual processing pathways and cognitive dysfunction (Murueta-Goyena et al., 2023).

Parallel to cognitive decline, neuropsychiatric symptoms—particularly depression, anxiety, apathy, and irritability—are prevalent across all stages of HD. Depression has been consistently associated with increased suicide risk, highlighting the need for early psychiatric monitoring and intervention (Duff et al., 2007; Hendel et al., 2021; Hubers et al., 2013). Furthermore, symptoms such as apathy and emotional blunting are increasingly recognized in pre-manifest individuals and those with reduced penetrance alleles, raising concern over the heterogeneity and unpredictability of disease onset (Abdollah Zadegan et al., 2023; Quarrell et al., 2007). Therefore, identifying factors associated with increased suicide ideation, particularly during the presymptomatic stages, is crucial. Nonetheless, the lack of reliable tools to diagnose prodromal symptoms presents a challenge for early intervention (Paulsen and Long, 2014).

Reduced penetrance (RP) alleles (who are individuals with expansions between 36 and 39 CAG repeats that may or may not develop HD) represent a unique subgroup, as some carriers develop clinical HD while others remain asymptomatic. Although motor status is often preserved, cognitive and psychiatric symptoms may still emerge, potentially signaling early disease progression (McDonnell et al., 2021; Quarrell et al., 2007). Understanding the clinical trajectory of RP carriers is therefore critical for refining prognostic models and informing therapeutic timing.

The main objective of this study was to longitudinally assess changes in visual cognition across the HD spectrum—including individuals with RP alleles—over a 1 year follow-up, and to determine its potential as an early marker of disease progression. In order to provide a broader clinical context and to explore factors that may influence disease trajectories, we also defined several secondary and exploratory objectives. The secondary objectives were: (i) to examine changes in motor status, general cognitive function, and neuropsychiatric symptoms (particularly apathy, depression, anxiety, and suicidal ideation) over time in HD gene carriers, including pre-manifest RP individuals; and (ii) to identify baseline predictors—such as premorbid intelligence, QoL, and apathy—that may be associated with more favorable or unfavorable progression. Exploratory objectives included: (i) characterizing the clinical course of RP individuals in comparison to other HD mutation carriers (pre-manifest, early manifest, and manifest stages). We hypothesized that (Ross et al., 2014) visual cognition would decline earlier and more sensitively than other cognitive domains, even in pre-manifest individuals (Hubers et al., 2013); higher premorbid intelligence, better baseline functioning, and lower baseline levels of neuropsychiatric symptoms would be associated with more favorable clinical trajectories; and (3) despite being motorically asymptomatic, RP individuals would show a distinct progression pattern over time compared to HC.

## 2 Materials and methods

### 2.1 Study design and participants

A total of 181 participants were recruited at baseline, divided into five groups according to disease duration and the presence or absence of motor symptoms: 40 pre-manifest HD, 30 early manifest HD (< 5 years from the diagnose), 27 manifest HD (> 5 years from the diagnose), six pre-manifest RP, and 78 HC, of which 142 participants were evaluated at 1 year follow-up (34 pre-manifest HD, 25 early manifest HD, 18 manifest HD, six with RP, and 59 HC). The RP group consists of participants with DNA results showing a CAG repeat range of 36–39 CAG.

Study subjects were recruited in the Department of Neurology of Cruces University Hospital, Araba University Hospital, and Donostia University Hospital (Spain). The inclusion criteria were the following: (1) Age > 18 years old; (2) Voluntary participation and signing the informed consent form; (3) Being able to read and write; (4) Specific inclusion criteria for HD carriers: having a genetic test that indicates the number of CAG repeats; (5) For the RP group, individuals were required not to present motor symptoms (UHDRS motor score within normal range) at the time of subject selection, ensuring that all participants were in a pre-manifest motor state at baseline. The exclusion criteria were as follows (1) Previous history of physical or mental disease external to HD (e.g., major psychiatric disorders, stroke, or other neurological diseases) that significantly compromised the cognitive function of the patient; (2) Visual or hearing limitation that could not be compensated with glasses or hearing aids; (3) History of alcohol or drug abuse meeting DSM-5 criteria for substance use disorder in the past 12 months, or a pattern of use that, in the investigator’s judgment, could interfere with cognitive performance or study participation; (4) Lack of will or incapacity of the participant to collaborate with the study.

The study protocol was approved by the Basque Drug Research Ethics Committee [*Comité de Ética de la Investigación con medicamentos de Euskadi* (CEIm-E) (PI2020117)]. All participants gave written informed consent prior to their participation in the study, in accordance with the tenets of the Declaration of Helsinki. The recruitment period for this study was from 1 September 2020 to 31 December 2023.

### 2.2 Motor, neuropsychological, neuropsychiatric and general health assessment

The clinical evaluation protocol consisted of measuring the motor status, the neuropsychological, neuropsychiatric symptoms, and general health with a comprehensive evaluation. A full description of each neuropsychological test (constructs assessed, administration, scoring, and references) is provided in Supplementary Table 1. The specific variables included in each cognitive domain and retained for the analyses are listed in Supplementary Figure 1: (1) Motor status [Unified Huntington’s Disease Rating Scale-motor (UHDRS) (Kieburtz, 1996)]; (2) Neuropsychological assessment: General cognitive status [Montreal Cognitive Assessment (MoCA) (Nasreddine et al., 2005; Ojeda et al., 2016)]; Premorbid intelligence (Word Accentuation Test) (Del Pino et al., 2018), Verbal fluency [FAS word fluency test and letter P (Spreen and Benton, 1969)], Visual memory [Taylor Complex Figure (TCF) (del Pino et al., 2015) and the Brief Visuospatial Memory Test-Revised (BVMT-R) (Benedict et al., 1996; Sáez-Atxukarro et al., 2021)], Visual Processing speed [Salthouse Perceptual Comparison Test (SPCT) (Peña et al., 2016; Salthouse, 1991) and Symbol Digit Modalities test (SDMT) (Peña-Casanova et al., 2009; Smith, 2007)], Visual Attention [Stroop Test (Word, Color, Word-Color) (Golden, 2001) and Trail Making Test (TMT) (Reitan and Wolfson, 1985) part A], Executive function {Modified Wisconsin Card Sorting Test [M-WCST (del Pino et al., 2016; Schretlen, 2010)] and TMT part B}, Visuospatial abilities [Benton Judgment of Line Orientation (BJLO) (Benton et al., 1975; Benton et al., 1994), Visuospatial abilities with Visual Object and Space Perception Battery (VOSP) (Warrington and James, 1991) cubes and dot counting, and Grooved Pegboard test (GPT)]; (Hoogeveen et al., 1993) Neuropsychiatric symptoms: Anxiety and depression [Hospital Anxiety and Depression Scale (HADS)], Irritability scale (Snaith), Apathy [Lille apathy rating scale (LARS)], 3.4. Suicide risk [Columbia Suicide Severity Rating Scale (C-SSRS)]; (4) General health: Patient Health Questionnaire (PHQ-9), QoL [Scale of QoL (GENCAT)], and Activities of daily living [Instrumental Activities of daily living (IADL)].

The scores of TMT-A, TMT-B, GPT time, M-WCST errors, M-WCST perseverative errors, PHQ-9, HADS, LARS, and C-SSRS were inverted in order to all the measures would be aligned in the same direction, with higher scores reflecting better performance or lower impairment. This transformation ensured consistency across domains, as the original raw scores varied in direction. All variables were subsequently standardized (Z-scores), and cognitive composites were created by averaging the Z-scores of the relevant cognitive tests within each domain. Z-scores were calculated for both baseline and 1-year follow-up visits. Confirmatory factor analysis (CFA) was performed to verify the construct validity of the six cognitive domains (visual attention, visual processing speed/visual perception, visuospatial abilities, visual memory, executive function, and verbal fluency); the exact mapping of variables to domains is provided in Supplementary Figure 1. The model presented adequate fit values: Comparative Fit Index (CFI) = 0.90, Tucker-Lewis Index (TLI) = 0.88, and Root Mean Square error of approximation = 0.098 (Pino et al., 2025).

### 2.3 Statistical analysis

Statistical analyses were performed using R software (version 4.3.1) and IBM SPSS Statistics for Windows, version 20.0 (IBM-SPSS, Armonk, New York). The assumptions of normality and homogeneity of variances of the variables were analyzed. The normality of continuous variables was assessed using the Shapiro-Wilk test. Mean and standard deviation are reported if the distribution was normal, or median and interquartile range otherwise. Group comparisons were conducted using either ANOVA or Kruskal-Wallis test, depending on the distributional characteristics of the variable. Categorical variables are presented as frequency and percentage. Group comparisons for categorical variables were performed using either the Chi-square test or Fisher’s exact test when the expected count was less than 5.

Linear mixed-effect models (LMMs) were employed to analyze differences in z-scores between the two visits. Motor status (UHDRS), cognitive and neuropsychiatric domains, and general health variables were used as outcome variables. The following fixed effects were included in LMMs: group, visit [visit 1 (baseline) and visit 2 (1 year follow-up)], group*visit, sex, age at baseline, education, affected progenitor (mother/father), and premorbid intelligence at baseline (Word Accentuation Test, WAT). HC group was used as the reference group. In addition, two sub-analyses excluding the HC group was conducted. For these LMMs, the following fixed effects were introduced: group, visit [visit 1 (baseline) and visit 2 (1 year follow-up)], sex, age, education, affected progenitor (mother/father), premorbid intelligence, CAG repeats, time since symptom onset (years), and years to estimated onset (YTO). YTO was derived from the exponential model proposed by Langbehn et al. (2004), where the age at onset (AAO) is estimated as:

AAO = 21.54 + exp (9.556–0.146 × CAG). Therefore, YTO corresponds to the difference between AAO and the participant’s chronological age. In all models, for motor and cognitive outcomes, neuropsychiatric variables and daily functioning, and QoL at baseline were included as predictors. A random intercept for subjects was included as random effects. Non-significant predictors were excluded one by one until the optimal model with significant variables was obtained. Conditional R^2^ is reported for each model. As commonly observed in longitudinal studies, some loss to follow-up occurred between baseline and the 1-year follow-up. Participants who did not complete the follow-up were mainly lost due to personal reasons (e.g., changes in residence, increased caregiver burden, loss of interest), health-related issues preventing further participation, or voluntary withdrawal. Importantly, longitudinal analyses were conducted using linear mixed-effect models, which can appropriately handle unbalanced data and missing observations under the assumption of missing at random.

## 3 Results

The general clinical characteristics of participants are described in Table 1. The mean age was 49.9 years, being pre-manifest patients significantly younger than other groups; 56% of the participants were woman. The group of HD carriers (pre-manifest, early manifest and manifest) had a lower premorbid intelligence than the RP and HC groups. There were no significant differences between pre-manifest, early manifest and manifest HD patients in the number of CAG repeats. 61.9% of patients inherited the mutation from their mother. As expected, the manifest HD group had higher motor impairment in the UHDRS (41.3 ± 21.6) than the early manifest HD patients (25 ± 14.1; *p* < 0.001) at inclusion. These baseline characteristics were considered in the subsequent regression models to account for their potential influence on the longitudinal progression of clinical and cognitive outcomes.

**TABLE 1:** Sociodemographic and clinical data.

	All *N* = 181	A (HC) *n* = 78	B (RP) *n* = 6	C (pre-manifest) n = 40	D (early manifest) *n* = 30	E (manifest) n = 27	*P*	*Post hoc* analyses								
								AvsC	AvsD	AvsE	BvsC	BvsD	BvsE	CvsD	CvsE	DvsE
Sex (men), *n* (%)	80 (44%)	35 (44.9)	3 (50%)	16 (40%)	11 (36.7%)	15 (53.6%)	–	–	–	–	–	–	–	–	–	–
Age (yrs)	49.9 (12.1)	50.8 (12.8)	52.0 (12.4)	43.0 (11.6)	51.6 (7.2)	54.9 (11.3)	0.001	**	–	–	–	–	–	*	***	–
Education (yrs)	12.7 (3.6)	13.1 (4.0)	13.7 (1.8)	12.8 (3.4)	12.6 (3.0)	11.7 (3.7)	–	–	–	–	–	–	–	–	–	–
Premorbid intelligence	23.3 (4.5)	24.6 (3.9)	24.5 (3.3)	23.0 (3.9)	22.6 (3.6)	20.5 (6.6)	0.001	–	–	***	–	–	–	–	–	–
CAG repeats	42.5 (2.9)	–	36.8 (1.8)	42.5 (2.3)	42.5 (2.1)	43.9 (3.1)	< 0.001	–	–	–	***	***	***	–	–	–
Age at first symptom (yrs)	47.1 (8.3)	–	–	47.1 (8.2)	48.9 (7.6)	44.9 (9.1)	–	–	–	–	–	–	–	–	–	–
Time to first symptom (yrs)	1.6 (3.2)	–	–	0.6 (1.6)	3.0 (2.7)	6.8 (4.4)	< 0.001	–	***	***	–	*	***	***	***	***
AAO	52.7 (13.9)	–	88.7 (17.2)	51.5 (8.9)	51.5 (8.7)	47.4 (11.8)	< 0.001	–	–	–	***	***	***	–	–	–
YTO	3.9 (14.5)	–	36.7 (14.8)	8.9 (9.8)	−0.04 (9.5)	−7.0 (9.6)	< 0.001	–	–	–	***	***	***	**	***	–
**Parent affected, *n* (%)**																
Father	40 (38.1%)	–	4 (66.7%)	11 (29.7%)	16 (53.3%)	7 (26.9%)	–	–	–	–	–	–	–	–	–	–
Mother	65 (61.9%)	–	2 (33.3%)	26 (70.3%)	14 (46.7%)	19 (73.1%)	–	–	–	–	–	–	–	–	–	–
**Baseline**																
Motor status	10.9 (18.8)	–	1.40 (1.5)	1.48 (2.2)	25.0 (14.1)	41.3 (21.6)	< 0.001	–	***	***	–	***	***	***	***	***
General cognition	24.4 (4.7)	26.3 (3.4)	27.3 (3.7)	25.9 (3.0)	21.8 (4.9)	18.9 (4.7)	< 0.001	–	***	***	–	*	***	***	***	*
Verbal Fluency	−0.02 (0.9)	0.37 (0.7)	0.73 (0.5)	0.11 (0.7)	−0.54 (0.7)	−0.86 (0.9)	< 0.001	–	***	***	–	**	***	**	***	–
Executive function	0.03 (0.8)	0.33 (0.4)	0.40 (0.5)	0.22 (0.7)	−0.35 (0.8)	−1.02 (0.7)	< 0.001	–	***	***	–	–	***	*	***	*
Visual memory	–0.02 (0.9)	0.40 (0.7)	0.86 (0.7)	0.22 (0.7)	−0.77 (0.6)	−1.03 (0.5)	< 0.001	–	***	***	–	***	***	***	***	–
Visuospatial abilities	0.01 (0.7)	0.37 (0.3)	0.27 (0.5)	0.30 (0.3)	−0.51 (0.6)	−0.99 (0.7)	< 0.001	–	***	***	–	**	***	***	***	**
Visual attention	−0.02 (0.5)	0.18 (0.4)	0.11 (0.5)	0.20 (0.4)	−0.36 (0.3)	−0.57 (0.6)	< 0.001	–	***	***	–	–	*	***	***	–
Visual processing speed/visual perception	–0.04 (0.9)	0.34 (0.7)	0.13 (0.7)	0.36 (0.7)	0.68 (0.7)	1.18 (0.7)	< 0.001	–	***	***	–	–	***	***	***	–
Anxiety and depression	10.2 (9.0)	8.47 (6.4)	9.00 (5.4)	10.7 (8.7)	11.9 (13.5)	13.0 (10.1)	–	–	–	–	–	–	–	–	–	–
Irritability	8.70 (7.7)	7.82 (6.5)	10.0 (9.4)	9.30 (8.3)	7.80 (7.8)	11.0 (9.0)	–	–	–	–	–	–	–	–	–	–
Apathy	−24.01 (12.4)	−28.12 (6.5)	−29.17 (5.4)	−25.27 (10.9)	−20.37 (15.4)	−13.33 (16.9)	< 0.001	–	*	***	–	–	*	–	***	–
Suicide ideation	1.51 (4.8)	1.06 (4.0)	4.33 (9.6)	0.62 (2.4)	2.33 (6.6)	2.58 (5.8)	–	–	–	–	–	–	–	–	–	–
General health	−0.02 (1.0)	0.07 (0.7)	−0.39 (1.1)	0.12 (0.9)	−0.08 (1.3)	−0.34 (1.2)	–	–	–	–	–	–	–	–	–	–
QoL	251 (23.5)	256 (15.4)	254 (18.1)	252 (22.8)	252 (22.6)	234 (35.9)	0.001	–	–	***	–	–	–	–	**	*
Daily life activities	7.44 (1.5)	7.90 (0.5)	7.83 (0.4)	7.97 (0.1)	7.47 (1.2)	5.22 (2.8)	< 0.001	–	–	***	–	–	***	–	***	***
**1-Year follow-up**																
Motor status	10.6 (19.9)	–	2.83 (4.6)	3.04 (4.0)	30.8 (14.6)	48.5 (24.5)	< 0.001	–	***	***	–	***	***	***	***	***
General cognition	24.4 (4.7)	26.4 (2.7)	27.0 (2.6)	26.3 (2.7)	21.2 (4.5)	18.3 (5.7)	< 0.001	–	***	***	–	**	***	***	***	–
Verbal Fluency	−0.04 (0.9)	0.34 (0.6)	0.57 (0.7)	0.27 (0.7)	−0.72 (0.7)	−1.09 (0.7)	< 0.001	–	***	***	–	***	***	***	***	–
Executive function	0.01 (0.8)	0.31 (0.4)	0.37 (0.5)	0.20 (0.7)	−0.38 (0.8)	−1.07 (0.7)	< 0.001	–	***	***	–	–	***	*	***	*
Visual memory	−0.04 (0.7)	0.31 (0.6)	0.33 (0.3)	−0.04 (0.5)	−0.46 (0.7)	−0.72 (0.5)	< 0.001	–	***	***	–	–	**	–	**	–
Visuospatial abilities	−0.01 (0.6)	0.27 (0.2)	0.22 (0.2)	0.24 (0.3)	−0.38 (0.5)	−0.97 (0.8)	< 0.001	–	***	***	–	*	***	***	***	***
Visual attention	−0.02 (0.8)	0.36 (0.5)	0.12 (0.5)	0.38 (0.7)	−0.65 (0.6)	−1.25 (0.8)	< 0.001	–	***	***	–	–	***	***	***	*
Visual processing speed/visual perception	−0.05 (0.8)	0.34 (0.6)	0.03 (0.7)	0.38 (0.7)	−0.77 (0.6)	−1.17 (0.5)	< 0.001	–	***	***	–	–	**	***	***	–
Anxiety and depression	9.97 (8.0)	9.00 (7.0)	11.8 (8.0)	9.03 (7.4)	11.1 (10.6)	12.6 (8.5)	–	–	–	–	–	–	–	–	–	–
Irritability	7.81 (7.0)	7.64 (5.7)	9.50 (9.4)	5.91 (7.5)	8.21 (7.6)	10.6 (8.0)	–	–	–	–	–	–	–	–	–	–
Apathy	−22.64 (12.4)	−26.40 (8.5)	−21.67 (16.4)	−22.35 (15.2)	−21.58 (10.4)	−12.95 (14.0)	0.001	–	–	***	–	–	–	–	**	–
Suicide ideation	0.56 (2.7)	0.27 (1.7)	3.00 (7.3)	0.00 (0.0)	0.36 (1.2)	2.00 (5.4)	0.019	–	–	–	–	–	–	–	–	–
General Health	−0.02 (1.0)	0.07 (0.7)	−0.39 (1.1)	0.12 (0.9)	−0.08 (1.3)	−0.34 (1.2)	–	–	–	–	–	–	–	–	–	–
QoL	249 (29.6)	253 (19.8)	255 (16.9)	246 (45.8)	248 (26.0)	242 (26.2)	–	–	–	–	–	–	–	–	–	–
Daily life activities	7.51 (1.4)	7.98 (0.1)	7.83 (0.4)	7.74 (1.3)	7.52 (1.3)	5.47 (2.4)	< 0.001	–	–	***	–	–	***	–	***	***

*** ≤ 0.001; ** ≤ 0.01; * ≤ 0.05. The *p*-values > 0.05 were deleted from the table. AAO, estimated age at onset; HC, healthy controls; RP, reduced penetrance; QoL, quality of life; YTO, years to estimated onset. instruments: Motor status (UHDRS-motor part); General cognition [Montreal Cognitive Assessment (MoCA)]; Verbal Fluency (FAS word fluency test); Executive function [Modified Wisconsin Card Sorting Test (M-WCST) and TMT part B]; Visuospatial abilities [Benton Judgment of Line Orientation (BJLO)]; Visual attention [Stroop Test (Word, Color, Word-Color) and Trail Making Test (TMT) part A]; Visual processing speed/visual perception [Salthouse Perceptual Comparison Test (SPCT)]; Anxiety and depression: Hospital Anxiety and Depression Scale (HADS)]; Apathy: Lille apathy rating scale (LARS); Suicide: Columbia Suicide Severity Rating Scale (C-SSRS); General health [Patient Health Questionnaire (PHQ-9)]; QoL: Quality of Life (GENCAT); IADL: Instrumental activities of daily living.

In line with our primary objective, we first examined longitudinal changes in visual cognition across the HD spectrum, including RP individuals. Subsequently, we explored motor status, general cognition, and neuropsychiatric symptom progression as secondary outcomes, and evaluated potential predictors of clinical trajectories. Finally, exploratory analyses focused on pre-manifest and RP-specific patterns and their relationship to other HD subgroups and symptom domains.

**FIGURE 1:**
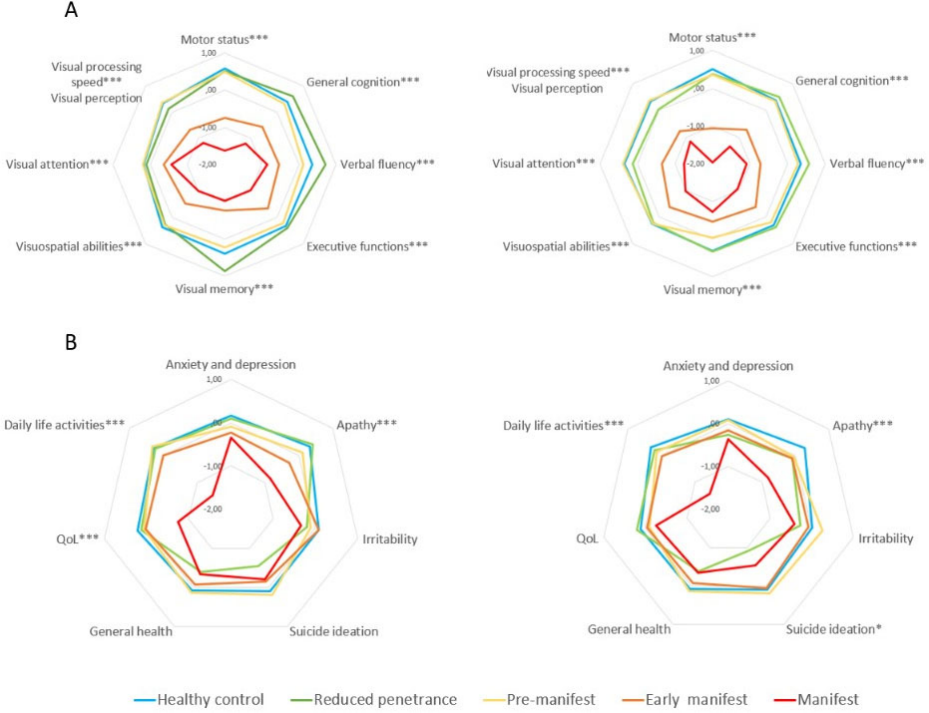
Neuropsychological and neuropsychiatric differences between groups at baseline and 1 year follow-up. **(A)** Motor status and neuropsychological performance. **(B)** Neuropsychiatric symptoms and general health. *** ≤ 0.001; ** ≤ 0.01; * ≤ 0.05. Instruments: Motor status (UHDRS-motor part); General cognition [Montreal Cognitive Assessment (MoCA)]; Verbal Fluency (FAS word fluency test); Executive function [Modified Wisconsin Card Sorting Test (M-WCST) and TMT part B]; Visuospatial abilities [Benton Judgment of Line Orientation (BJLO)]; Visual attention [Stroop Test (Word, Color, Word-Color) and Trail Making Test (TMT) part A]; Visual processing speed/visual perception [Salthouse Perceptual Comparison Test (SPCT)]; Anxiety and depression: Hospital Anxiety and Depression Scale (HADS)]; Apathy: Lille apathy rating scale (LARS); Suicide: Columbia Suicide Severity Rating Scale (C-SSRS); General health [Patient Health Questionnaire (PHQ-9)]; QoL: Quality of Life (GENCAT); IADL: Instrumental activities of daily living.

At one-year follow-up, visual cognition emerged as a particularly sensitive domain for early decline across the HD spectrum. Significant worsening in visual memory and visual attention was observed in pre-manifest and RP participants, with additional impairments in visual processing speed and verbal fluency specifically noted in the RP group—changes that were not present in HC (Figure 1 and Table 1). These findings highlight the potential utility of visual cognitive measures as early clinical markers of disease progression, even in individuals without motor symptoms.

Motor performance also declined significantly in all clinical HD groups, with the most pronounced deterioration occurring in early manifest and manifest patients (Figures 1A, B). A milder, non-significant trend toward motor decline was noted in the pre-manifest and RP groups. General cognitive decline was also evident, particularly in early and manifest HD stages, whereas pre-manifest individuals exhibited more subtle changes. The RP group showed preservation of global cognition overall, despite selective deterioration in specific visual domains. In parallel, neuropsychiatric symptoms worsened over time across HD mutation carriers (*p* < 0.01; Table 1), with manifest patients showing significant increases in apathy, anxiety, depression, and irritability, as well as marked reductions in daily functioning and QoL. Notably, the RP group—despite being motorically asymptomatic—presented elevated irritability and the highest levels of suicidal ideation at follow-up, although this difference did not reach statistical significance. These findings reflect a pattern of progressive multisystem involvement and underscore the importance of early detection and monitoring beyond motor manifestations, particularly in at-risk subgroups.

### 3.1 Longitudinal modeling of domain-specific trajectories and predictors of progression

**FIGURE 2:**
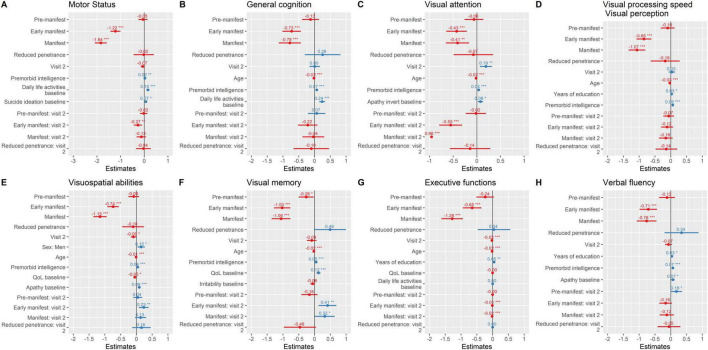
Motor status and neuropsychological performance over time. Parameter estimates from linear mixed-effects models are plotted. **(A)** Motor status; **(B)** General Cognition; **(C)** Visual attention; **(D)** visual processing speed/visual perception; **(E)** visuospatial abilities; **(F)** Visual memory; **(G)** Executive functions; **(H)** Verbal fluency. *** ≤ 0.001; ** ≤ 0.01; * ≤ 0.05. Instruments: Motor status (UHDRS-motor part); General cognition [Montreal Cognitive Assessment (MoCA)]; Verbal Fluency (FAS word fluency test); Executive function [Modified Wisconsin Card Sorting Test (M-WCST) and TMT part B]; Visuospatial abilities [Benton Judgment of Line Orientation (BJLO)]; Visual attention [Stroop Test (Word, Colour, Word-Colour) and Trail Making Test (TMT) part A]; Visual processing speed/visual perception [Salthouse Perceptual Comparison Test (SPCT)]; Anxiety and depression: Hospital Anxiety and Depression Scale (HADS)]; Apathy: Lille apathy rating scale (LARS); Suicide: Columbia Suicide Severity Rating Scale (C-SSRS); General health [Patient Health Questionnaire (PHQ-9)]; QoL: Quality of Life (GENCAT); IADL: Instrumental activities of daily living.

To further characterize domain-specific progression and test our hypotheses regarding predictors of clinical outcomes, we applied LMMs including group*visit interactions and comparing HD spectrum with HC (Figure 2, Table 2, and Supplementary Table 2). Visual cognition was consistently the first domain to show early and significant alterations across the disease spectrum. Visual attention, processing speed/perception, visuospatial abilities, visual memory, executive functions, and verbal fluency all declined in mutation carriers compared with controls (Figures 2C–H), with the steepest deterioration observed in early manifest (β range = −0.43 to −1.0, all *p* < 0.001) and manifest patients (β range = −0.41 to −1.3, all *p* < 0.001). Pre-manifest individuals showed subtle but significant decline in visual memory (β = −0.26, *p* = 0.033), while the RP group did not exhibit consistent cognitive deterioration, although trends toward worsening were noted in several visual domains. Interaction analyses indicated accelerated decline at follow-up in early manifest carriers for visual attention (β = −0.55, *p* < 0.001), visuospatial abilities (β = 0.23, *p* = 0.004), and visual memory (β = 0.41, *p* = 0.005), as well as in manifest carriers for visual attention (β = −0.96, *p* < 0.001) and visual memory (β = 0.32, *p* = 0.045). These findings reinforce the sensitivity of visual cognition to early disease progression.

**TABLE 2:** Linear mixed-effect models.

Linear mixed-effect models comparing HD carriers with healthy controls	AIC	R^2^
**Motor function**		
UHDRS ∼ 1 + group + visit + premorbid intelligence + IADL + C-SSRS + Group*Visit + (1 | id)	385.0	0.94
**Cognition**		
MoCA ∼ 1 + group + visit + age + premorbid intelligence + IADL + Group*Visit + (1 | id)	679.33	0.77
Visual attention ∼ 1 + group + visit + age + premorbid intelligence + Apathy, + Group*Visit + (1 | id)	475.2	0.75
Visual processing speed/visual perception ∼ 1 + group + visit + age + premorbid intelligence + Group*Visit + (1 | id)	458.6	0.90
Visuospatial abilities ∼ 1 + group + visit + age + premorbid intelligence + QoL + Apathy + Group*Visit + (1 | id)	328.6	0.88
Visual memory ∼ 1 + group + visit + age + premorbid intelligence + QoL + Irritability + Group*Visit + (1 | id)	601.7	0.75
Executive function ∼ 1 + group + visit + age + premorbid intelligence + QoL + IADL + Group*Visit + (1 | id)	−475.7	1
Verbal fluency ∼ 1 + group + visit + years of education + premorbid intelligence + apathy + Group*Visit + (1 | id)	555.1	0.88
**Neuropsychiatric symptoms**		
Anxiety and depression ∼ 1 + group + visit + sex + age + Group*Visit + (1 | id)	905.8	0.46
Irritability ∼ 1 + group + visit + sex + years of education + Group*Visit + (1 | id)	889.0	0.61
Apathy ∼ 1 + group + visit + years of education + Group*Visit + (1 | id)	889.3	0.40
Suicide ∼ 1 + group + visit + Group*Visit + (1 | id)	854.5	0.63
**General health**		
QoL ∼ 1 + group + visit + Group*Visit + (1 | id)	923.2	0.41
Daily functioning ∼ 1 + group + visit + parent affected + premorbid intelligence + Group*Visit + (1 | id)	547.8	0.71
**Sub-study excluding healthy controls and using Pre-manifest group as reference**		
**Motor function**		
UHDRS ∼ 1 + group + visit + premorbid intelligence + IADL + C-SSRS + YTO + Group*Visit + (1 | id)	293.1	0.92
**Cognition**		
MoCA ∼ 1 + group + visit + age + premorbid intelligence + IADL + Group*Visit + (1 | id)	378.4	0.86
Visual attention ∼ 1 + group + visit + age + premorbid intelligence + Apathy + YTO + Group*Visit + (1 | id)	319.2	0.73
Visual processing speed/visual perception ∼ 1 + group + visit + age + years of education + premorbid intelligence + YTO + Group*Visit + (1 | id)	297.6	0.91
Visuospatial abilities ∼ 1 + group + visit + age + premorbid intelligence + QoL + Apathy + IADL + Group*Visit + (1 | id)	248.5	0.87
Visual memory ∼ 1 + group + visit + age + premorbid intelligence + QoL + Irritability + Group*Visit + (1 | id)	320.0	0.77
Executive function ∼ 1 + group + visit + age + premorbid intelligence + QoL + IADL + Group*Visit + (1 | id)	−166.0	1
Verbal fluency ∼ 1 + group + visit + years of education + premorbid intelligence + QoL + Apathy + YTO + Group*Visit + (1 | id)	393.9	0.91
**Neuropsychiatric symptoms**		
Anxiety and depression ∼ 1 + group + visit + sex + age + Group*Visit + (1 | id)	562.3	0.36
Irritability ∼ 1 + group + visit + sex + years of education + Group*Visit + (1 | id)	543.3	0.56
Apathy ∼ 1 + group + visit + years of education + Group*Visit + (1 | id)	565.9	0.29
Suicide ∼ 1 + group + visit + Group*Visit + (1 | id)	535.2	0.60
**General health**		
QoL ∼ 1 + group + visit + Group*Visit + (1 | id)	585.1	0.35
Daily functioning ∼ 1 + group + visit + parent affected + premorbid intelligence + Group*Visit + (1 | id)	506.1	0.70
**Sub-study (comparing HD carriers with Reduced penetrance group)**		
**Motor function**		
UHDRS ∼ 1 + group + visit + age + premorbid intelligence + CAG repeats + time to first symptom + IADL + Group*Visit + (1 | id)	299.1	0.92
**Cognition**		
MoCA ∼ 1 + group + visit + age + years of education + premorbid intelligence + IADL + Group*Visit + (1 | id)	378.2	0.86
Visual attention ∼ 1 + group + visit + age + premorbid intelligence + Apathy, + YTO + Group*Visit + (1 | id)	319.2	0.73
Visual processing speed/visual perception ∼ 1 + group + visit + age + premorbid intelligence + CAG repeats + Group*Visit + (1 | id)	293.6	0.91
Visuospatial abilities ∼ 1 + group + visit + age + premorbid intelligence + time to first symptom + QoL + Apathy + IADL + Group*Visit + (1 | id)	250.6	0.87
Visual memory ∼ 1 + group + visit + age + premorbid intelligence + CAG repeats + QoL + Irritability + Group*Visit + (1 | id)	321.5	0.77
Executive function ∼ 1 + group + visit + age + education + time to first symptom + Group*Visit + (1 | id)	−197.9	1
Verbal fluency ∼ 1 + group + visit + years of education + premorbid intelligence + CAG repeats + QoL + apathy + Group*Visit + (1 | id)	327.2	0.91
**Neuropsychiatric symptoms**		
Anxiety and depression ∼ 1 + group + visit + sex + age + Group*Visit + (1 | id)	562.3	0.36
Irritability ∼ 1 + group + visit + years of education + Group*Visit + (1 | id)	544.7	0.56
Suicide ∼ 1 + group + visit + CAG repeats + Group*Visit + (1 | id)	538.6	0.57
**General health**		
QoL ∼ 1 + group + visit + education + Group*Visit + (1 | id)	587.5	0.35
Daily functioning ∼ 1 + group + visit + parent affected + premorbid intelligence + Group*Visit + (1 | id)	506.1	0.70

Age, age at baseline; YTO, years to estimated onset. Instruments: Motor status (UHDRS-motor part); General cognition [Montreal Cognitive Assessment (MoCA)]; Verbal Fluency (FAS word fluency test); Executive function [Modified Wisconsin Card Sorting Test (M-WCST) and TMT part B]; Visuospatial abilities [Benton Judgment of Line Orientation (BJLO)]; Visual attention [Stroop Test (Word, Colour, Word-Colour) and Trail Making Test (TMT) part A]; Visual processing speed/visual perception [Salthouse Perceptual Comparison Test (SPCT)]; Anxiety and depression: Hospital Anxiety and Depression Scale (HADS)]; Apathy: Lille apathy rating scale (LARS); Suicide: Columbia Suicide Severity Rating Scale (C-SSRS); General health [Patient Health Questionnaire (PHQ-9)]; QoL, quality of life (GENCAT); IADL, instrumental activities of daily living.

Across these cognitive domains, age consistently emerged as a negative predictor, whereas premorbid intelligence (β range = 0.04–0.07, all *p* < 0.001) and years of education acted as protective factors in several models. Baseline apathy was associated with poorer trajectories in visual attention, visuospatial abilities, and verbal fluency, while lower QoL and irritability contributed to worse outcomes in visuospatial abilities and visual memory, respectively. Together, these results support the role of visual and executive domains as early and clinically relevant markers of disease progression, highlighting both cognitive reserve and neuropsychiatric symptoms as modulators of decline.

Motor performance showed significant longitudinal decline across groups, with the steepest deterioration observed in early manifest (β = −1.2, *p* < 0.001) and manifest carriers (β = −1.8, *p* < 0.001). In contrast, pre-manifest and RP individuals exhibited only mild, non-significant trends (β = −0.05 and −0.02 points/year, respectively) (Figure 2A). The interaction analyses further indicated that the rate of decline was particularly pronounced in early manifest carriers at follow-up (group*visit β = −0.27, *p* = 0.003). In addition, premorbid intelligence (β = 0.02, *p* = 0.004) and baseline daily life activities (β = 0.15, *p* < 0.001) emerged as protective factors, whereas baseline suicidal ideation predicted poorer trajectories (β = 0.07, *p* = 0.013). General cognition followed a similar pattern (Figure 2B), with significant decline in early manifest (β = −0.73, *p* < 0.001) and manifest carriers (β = −0.78, *p* < 0.001), while pre-manifest and RP individuals did not differ significantly from healthy controls (β = −0.13 and 0.26, respectively). No significant longitudinal change was detected across visits, and the interaction terms confirmed that the decline was not accelerated over time in any group. Age (β = −0.02, *p* < 0.001) was a negative predictor, while premorbid intelligence (β = 0.07, *p* < 0.001) and baseline daily life activities (β = 0.24, *p* < 0.001) emerged as protective factors.

**FIGURE 3:**
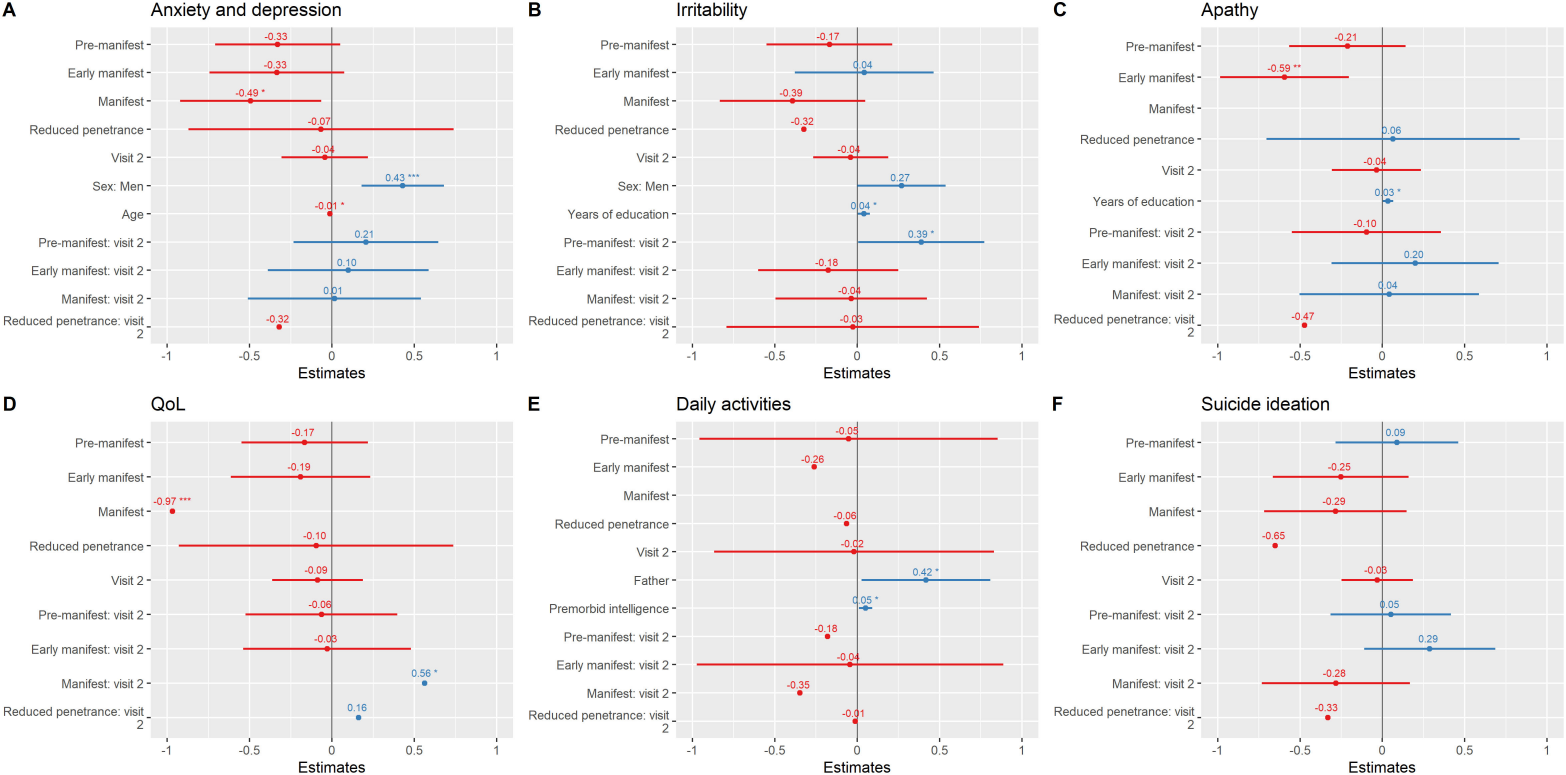
Neuropsychiatric symptomatology and general health variables over time. Parameter estimates from regression mixed models are plotted. **(A)** Anxiety and depression; **(B)** Irritability; **(C)** Apathy; **(D)** QoL; **(E)** Daily activities; **(F)** Suicide ideation. *** ≤ 0.001; ** ≤ 0.01; * ≤ 0.05. Instruments: Motor status (UHDRS-motor part); General cognition [Montreal Cognitive Assessment (MoCA)]; Verbal Fluency (FAS word fluency test); Executive function [Modified Wisconsin Card Sorting Test (M-WCST) and TMT part B]; Visuospatial abilities [Benton Judgment of Line Orientation (BJLO)]; Visual attention [Stroop Test (Word, Colour, Word-Colour) and Trail Making Test (TMT) part A]; Visual processing speed/visual perception [Salthouse Perceptual Comparison Test (SPCT)]; Anxiety and depression: Hospital Anxiety and Depression Scale (HADS)]; Apathy: Lille apathy rating scale (LARS); Suicide: Columbia Suicide Severity Rating Scale (C-SSRS); General health [Patient Health Questionnaire (PHQ-9)]; QoL: Quality of Life (GENCAT); IADL: Instrumental activities of daily living.

Neuropsychiatric and general health variables also worsened over time (Table 2, Figures 3A–F, and Supplementary Table 2). The manifest group showed the steepest decline, with significant worsening in anxiety and depression (β = −0.49, *p* = 0.024), irritability (β = −0.39, *p* = 0.08, trend), daily functioning (β = −1.6, *p* = 0.002), and QoL (β = −0.97, *p* < 0.001). Apathy increased significantly both in early manifest (β = −0.59, *p* = 0.003) and manifest carriers (β = −1.1, *p* < 0.001). The RP group did not show consistent changes across most domains but exhibited a marked, though non-significant, worsening in suicidal ideation (β = −0.65, *p* = 0.12), highlighting potential emotional vulnerability in this clinically silent subgroup. However, the results from the RP group should be interpreted cautiously given the small sample size.

Interaction analyses further indicated that pre-manifest individuals had a modest but significant increase in irritability at follow-up (group*visit β = 0.39, *p* = 0.047), while manifest carriers exhibited a longitudinal decline in QoL (group*visit β = 0.56, *p* = 0.047).

Several baseline protective factors were also identified. Higher premorbid intelligence, greater years of education, and better QoL at baseline predicted more stable trajectories, whereas higher baseline apathy was associated with poorer outcomes. Collectively, these models explained a large proportion of variance in motor (R^2^ = 0.94) and cognitive outcomes (R^2^ = 0.77–1), underscoring the role of cognitive reserve and emotional well-being as key modulators of disease course.

### 3.2 Exploratory analysis of progression patterns in pre-manifest HD group compared to other HD subgroups

To further characterize domain-specific progression and identify predictors of clinical outcomes, we applied LMMs excluding HC and including YTO as a covariate of the models. Results showed that YTO variable remain significant in the following models: motor function, visual attention, visual processing speed, and verbal fluency exhibited significant group differences and longitudinal changes across the HD spectrum using the pre-manifest group as a reference.

Regarding cognitive outcomes, visual memory showed the largest and most consistent decline across groups, with both early manifest (β = −0.80, *p* < 0.001) and manifest patients (β = −0.87, *p* < 0.001) performing worse than pre-manifest individuals. Longitudinally, visual memory declined further at follow-up (β = −0.27, *p* = 0.007), with strong group*visit interactions for early (β = 0.56, *p* < 0.001) and manifest carriers (β = 0.59, *p* < 0.001), while premorbid intelligence consistently emerged as a protective factor (β = 0.04, *p* = 0.001). Visual attention was also impaired at baseline in early manifest patients (β = −0.39, *p* = 0.009), with significant longitudinal decline confirmed by group*visit interactions in both early (β = −0.49, *p* = 0.002) and manifest carriers (β = −1.0, *p* < 0.001); emerging YTO as a significant predictor in this domain (β = 0.01, *p* = 0.024), underscoring the influence of disease proximity. Visual processing speed was highly sensitive to progression, with baseline impairments in early (β = −0.70, *p* < 0.001) and manifest carriers (β = −0.82, *p* < 0.001) and robust longitudinal decline (R^2^ = 0.913), while both premorbid intelligence (β = 0.06, *p* < 0.001) and YTO (β = 0.02, *p* = 0.006) predicted trajectories. Visuospatial abilities followed a similar pattern, with significant baseline differences in early (β = −0.66, *p* < 0.001) and manifest groups (β = −0.99, *p* < 0.001) and additional decline over time (group*visit interaction in early manifest: β = 0.21, *p* = 0.041). Executive functions showed marked impairment in manifest patients at baseline (β = −1.0, *p* < 0.001) and significant longitudinal decline across visits (β = −0.03, *p* < 0.001). Finally, verbal fluency was already reduced in early (β = −0.56, *p* = 0.001) and manifest carriers (β = −0.51, *p* = 0.012), with progressive deterioration over time (group*visit interactions: early β = −0.34, p = 0.003; manifest β = −0.33, *p* = 0.008), while both premorbid intelligence (β = 0.06, *p* < 0.001) and education (β = 0.05, *p* = 0.021) acted as protective factors. Taken together, these findings indicate that multiple visual domains and executive/fluency measures deteriorate progressively in HD mutation carriers, but visual memory stands out as the earliest and most vulnerable domain, while visual attention and processing speed provide complementary markers of ongoing decline strongly influenced by disease proximity and cognitive reserve.

For motor performance, early manifest (β = −0.98, *p* < 0.001) and manifest patients (β = −1.60, *p* < 0.001) showed the steepest deterioration relative to pre-manifest individuals, while the RP group also declined but with smaller magnitude (β = −0.73, *p* = 0.031). Longitudinally, motor decline was significant in manifest patients at the second visit (Grupo*Visit: β = −0.30 *p* = 0.029).

Regarding neuropsychiatric outcomes, QoL was significantly reduced in manifest patients compared with pre-manifest individuals (β = −0.88, *p* = 0.015), while no significant differences were found for early manifest or RP carriers. No clear longitudinal change was observed across visits, although manifest patients tended to show further decline. Apathy increased significantly in both early manifest (β = −0.59, *p* = 0.003) and manifest carriers (β = −1.1, *p* < 0.001). Anxiety and depression were also significantly higher in manifest patients compared to pre-manifest (β = −0.49, *p* = 0.024), and sex emerged as a relevant factor, with men reporting higher levels than women (β = 0.58, *p* = 0.004). Irritability showed a trend toward worsening in manifest carriers (β = −0.39, *p* = 0.081), with pre-manifest individuals exhibiting a modest but significant increase at follow-up (group*visit β = 0.39, *p* = 0.047). Suicidal ideation was markedly elevated in RP carriers, who showed significantly higher scores than pre-manifest individuals (β = −1.3, *p* = 0.033). Finally, activities of daily living were significantly impaired in manifest patients compared with pre-manifest individuals (β = −1.3, *p* < 0.001), while premorbid intelligence (β = 0.06, *p* = 0.014) and maternal inheritance of the mutation (β = 0.42, *p* = 0.053) were associated with better functional outcomes.

### 3.3 Exploratory analysis of progression patterns in RP carriers compared to other HD subgroups

To address our exploratory objective of characterizing the clinical course of individuals with RP alleles in comparison to other HD gene carriers, we performed a sub-analysis excluding HC. LMMs were also applied to directly compare RP participants with pre-manifest, early manifest, and manifest groups, focusing on motor, cognitive, and neuropsychiatric domains. Regarding general and visual cognitive performance, RP and pre-manifest HD individuals showed similar rates of decline in most domains, including general cognition, visual attention, visuospatial abilities, visual memory, and verbal fluency. In contrast, early and manifest groups exhibited significantly steeper deterioration across these domains relative to RP individuals, confirming that cognitive decline accelerates with clinical disease progression. Notably, visual processing speed/perception declined at a comparable rate in RP and HD carriers, suggesting that this domain may be particularly sensitive to early neuropathological changes, even before overt motor onset. Model fit was high, with R^2^ values ranging from 0.72 up to 1.00 across visual cognitive domains. Furthermore, in the model for visual attention, both YTO and premorbid intelligence emerged as significant predictors, highlighting the modulatory role of disease proximity and cognitive reserve in shaping cognitive trajectories.

In terms of motor function, and after adjusting for age, premorbid intelligence, CAG repeats, years from first symptom, and daily functioning, only the manifest group showed significantly worse motor performance compared to RP (β = −0.94, *p* = 0.033). Neuropsychiatric and general health outcomes revealed no significant differences between RP carriers and other HD subgroups in anxiety, irritability, apathy, activities of daily living, or QoL. In the anxiety model, however, male sex was associated with higher scores (β = 0.58, *p* = 0.004). Irritability was positively associated with years of education (β = 0.07, *p* = 0.031), while higher premorbid intelligence predicted better activities of daily living performance (β = 0.06, *p* = 0.014). Notably, pre-manifest group had significantly less suicidal ideation than RP (β = 1.50, *p* = 0.014), with early and manifest carriers also trending toward less values relative to RP (β = 1.2, *p* = 0.064 and β = 1.3, *p* = 0.060, respectively). No main effects of group*visit interactions were observed across outcomes, and YTO was not a significant predictor in these domains.

Given the small sample size of the RP group, these findings must be interpreted with caution. However, it is important to note that HD is a rare condition, and RP individuals represent an even more infrequent and under characterized subgroup. Despite the sample size limitation, our results underscore the clinical relevance of studying RP carriers, who may exhibit early signs of decline in visual and neuropsychiatric domains. Exploratory inspection of individual trajectories revealed substantial variability, with some RP participants maintaining stable profiles, while others displayed notable worsening in visual processing and emotional symptoms over one year. These individual profiles are presented in Supplementary Table 3 and highlight the need for ongoing longitudinal studies in this unique population.

## 4 Discussion

This study aimed to longitudinally assess changes in visual cognition across the HD spectrum—including pre-manifest individuals with RP alleles—over a one-year follow-up, and to determine its potential as an early marker of disease progression. Our findings reveal early decline in visual memory, visual attention, and processing speed/perception even among asymptomatic participants. These results confirm our central hypothesis that visual cognition constitutes an early and sensitive indicator of disease progression in HD mutation carriers, including those with RP alleles—despite being motorically asymptomatic, and reinforce its clinical value as a prodromal marker.

This longitudinal approach addresses two critical gaps in the HD literature: (1) the lack of longitudinal data on visual cognition in HD and its prodromal stages, and (2) the limited understanding of disease trajectories in preclinical stages. By clarifying these aspects, our findings contribute to the refinement of early detection strategies and may inform the development of timely, individualized interventions.

The literature focused previously on executive dysfunction being one of cognitive domains more impaired in initial stages of the disease (Paulsen and Long, 2014; Radulovich Muñoz et al., 2019; Verny et al., 2007). However, our results suggest that visual cognition—particularly visual memory, attention and processing speed—deteriorates earlier and more consistently, even in individuals without evident motor symptoms. This underscores the potential of these domains as sensitive indicators of early disease-related changes. Even the pre-manifest group presented a more impairment in visual memory compared to HC, and the RP group in visual processing speed/visual perception and visual attention, but not in executive function. While previous studies have explored the natural history of HD, and the timing of motor, cognitive and/or emotional symptom emergence (Paulsen and Long, 2014; Ross et al., 2014), to our knowledge, this is the first prospective study that focused on visual cognition impairment in early stages of the disease and asymptomatic HD carriers, including the RP group; and taking into account the premorbid intelligence and YTO as factors. Visual deficits are clinically important because they influence overall cognitive performance and have implications for daily functioning (Coppen et al., 2018; Labuschagne et al., 2016). Deficits in visual attention are linked to dysfunction in the parieto-occipital cortex, which integrates visual information and coordinates attention and spatial awareness. Damage to this area, part of the dorsal visual stream, can impair focus, visual processing, and attention shifting which is particularly important for many daily activities, such as driving, reading, and navigating complex environments (Coppen et al., 2018). Understanding the role of the parieto-occipital regions in visual attention deficits in HD and, specifically, in pre-clinical stages can help developing targeted cognitive and rehabilitation strategies aimed at improving patients’ QoL.

### 4.1 Disease progression across motor, cognition, and neuropsychiatric domains

As expected, HD mutation carriers exhibited progressive impairments across motor, general cognition, and neuropsychiatric domains, with early manifest and manifest patients being the most affected. However, we also observed domain-specific changes in pre-manifest HD and RP groups, particularly in visual cognition domains, suggesting subtle early dysfunction in these asymptomatic or minimally symptomatic individuals. Importantly, when analyses were restricted to HD subgroups (excluding HC), cognitive domains remained the earliest and most sensitive markers of decline, whereas motor changes were less evident in RP carriers. This pattern reinforces the view that cognitive decline—particularly in visual domains—precedes detectable motor impairment, challenging the traditional clinical thresholds for intervention and monitoring. Although motor disorders are often considered the most prominent feature in HD, our findings confirmed that non-motor symptoms such as cognitive deficits and neuropsychiatric disturbances appear earlier and are more pronounced in the initial stages of the disease (Hubers et al., 2013; Pino et al., 2025; Radulovich Muñoz et al., 2019; Redondo Vergé et al., 2001). Our results align with recent literature emphasizing the involvement of posterior brain regions and visual processing pathways in early HD pathology. These deficits, which affect visuomotor integration, navigation, and daily functioning, appear to emerge in parallel with neuropsychiatric symptoms—particularly suicidal ideation—even during preclinical stages. This pattern was especially evident in the RP group, which, despite being motorically asymptomatic at baseline, showed subtle deterioration in visual cognition and were the only subgroup presenting suicidal ideation at follow-up. These findings challenge the traditional view of RP carriers as clinically stable and underscore the need for closer monitoring and early intervention in this subgroup, reinforcing the value of visual cognitive and neuropsychiatric markers for detecting early disease progression in HD.

Neuropsychiatric symptoms—including apathy, anxiety, depression, irritability, and suicidal ideation—also worsened over time. Notably, the RP group exhibited the highest levels of suicidal ideation, reinforcing the idea that psychiatric symptoms can precede motor onset and should be actively monitored even in prodromal or low-penetrance cases. These findings align with prior studies highlighting the early emergence of psychiatric symptoms in HD (Duff et al., 2007; Eddy et al., 2016; Hubers et al., 2013). These symptoms—often underprioritized in HD research—worsened across all groups over the one-year follow-up and were strongly associated with motor and cognitive decline, particularly in manifest HD patients (Duff et al., 2007; Hubers et al., 2013). Psychiatric symptoms, which frequently emerge early or even before motor onset, remained among the most distressing aspects of the disease for both patients and caregivers (Duff et al., 2007; Eddy et al., 2016). Despite their clinical relevance, they often take a secondary role in HD management and research priorities (Eddy et al., 2016). In our study, apathy and suicidal ideation stood out as particularly significant. Depression and anxiety, the most prevalent symptoms, further increased the risk of suicidal ideation (Eddy et al., 2016). Strikingly, while higher suicidal ideation was expected in early manifest and pre-manifest individuals, the RP group emerged as the most vulnerable, showing the highest levels of suicidal ideation. Therefore, psychiatric risk may precede or parallel cognitive decline, and underscore the critical need for integrated psychiatric assessment and proactive intervention in pre-manifest individuals. Apathy also emerged as a key symptom in early manifest HD, potentially an early disease marker. It is linked to dysfunction in the mesolimbic pathway, disrupting dopamine signaling and affecting motivation and emotional regulation, even in preclinical phases (Abdollah Zadegan et al., 2023; Hendel et al., 2021). Understanding mesolimbic involvement in apathy is essential for developing targeted interventions. Apathy is well-known in Alzheimer’s and Parkinson’s, but its pathophysiology in HD is not well understood (Abdollah Zadegan et al., 2023).

Among the secondary objectives, we aimed to identify baseline predictors of disease progression. Although this study did not specifically aim to explore direct relationships between cognitive and neuropsychiatric domains, all neuropsychiatric variables were included as predictors in the longitudinal models of motor and cognitive outcomes. Notably, apathy, irritability, suicidal ideation, daily functioning, and QoL emerged as significant modulators of disease trajectories, particularly for motor and cognitive performance. These findings highlight the potential influence of neuropsychiatric status on clinical progression in HD and support the role of cognitive reserve and emotional wellbeing as protective factors (Coppen et al., 2018; Labuschagne et al., 2016). Specifically, higher premorbid intelligence, better baseline functioning, and greater QoL were associated with more favorable outcomes, while increased apathy and suicidal ideation at baseline predicted steeper decline. The lower premorbid intelligence presented in HD patients compared to RP group and HC, indicated a potential cognitive decline preceding the onset of HD symptoms.

Our longitudinal models excluding HC and using the pre-manifest group as reference further highlighted that visual memory, visual attention, visual processing speed, visuospatial abilities, and verbal fluency constitute the most sensitive markers of disease progression in HD mutation carriers. Among these, visual memory stood out as the earliest and most consistently affected domain, while attention and processing speed provided complementary markers of ongoing decline strongly influenced by disease proximity and cognitive reserve. Verbal fluency also deteriorated progressively, confirming its sensitivity to fronto-striatal pathology, whereas executive functions showed significant impairment only in manifest carriers (Henry and Crawford, 2004; Stout et al., 2011). Motor impairment was most evident in early manifest and manifest groups, with RP carriers showing a milder but detectable decline. Importantly, baseline premorbid intelligence and proximity to clinical onset significantly modulated trajectories across domains. YTO emerged as a robust predictor in visual attention, processing speed, and verbal fluency, while premorbid intelligence and education acted as protective factors across multiple domains. These findings align with prior work linking higher cognitive reserve to slower decline in HD and other neurodegenerative conditions (Fritz et al., 2016; Soloveva et al., 2018) and confirm YTO as a robust indicator of disease progression (Langbehn et al., 2004; Tabrizi et al., 2013).

Taken together, our results reinforce that cognitive changes, particularly in visual memory and visual attention, occur early and in parallel with motor dysfunction, and may serve as clinically relevant markers of progression in HD. The modulatory effects of premorbid intelligence and YTO highlight the need to incorporate both cognitive reserve proxies and disease burden indices into predictive models of HD trajectories. This integrative approach could refine clinical staging and support the development of individualized interventions aimed at preserving cognitive function and delaying disability (Rees et al., 2013).

By demonstrating that specific cognitive and motor domains decline even in the absence of overt motor symptoms, and that individual differences in reserve and disease burden significantly shape progression, our findings contribute to a growing body of evidence emphasizing the heterogeneity of HD trajectories. They also support the inclusion of visual and verbal cognitive measures, together with reserve-related factors, in longitudinal monitoring and trial design, particularly in the prodromal and early manifest stages.

Lastly, exploratory analyses of RP individuals highlighted the clinical relevance of monitoring RP carriers and revealed distinct patterns of change. RP participants showed a trajectory of decline largely similar to pre-manifest individuals across most cognitive domains, including general cognition, visual attention, visuospatial abilities, visual memory, and verbal fluency. However, visual processing speed/perception declined at a comparable rate in RP and other HD carriers, suggesting that this domain may be particularly sensitive to early neuropathological changes, even before motor onset. These findings challenge the assumption that RP carriers are clinically stable and underscore the need for closer monitoring. In terms of motor function, RP carriers did not differ from pre-manifest or early manifest individuals, reinforcing the idea that cognitive alterations, rather than motor changes, may be the earliest detectable signals in this subgroup. From a neuropsychiatric perspective, RP carriers emerged as the only subgroup presenting suicidal ideation, significantly more than pre-manifest individuals and with a tendency compared to early and manifest carriers. This distinct profile suggests that psychiatric vulnerability may accompany, or even precede, subtle cognitive decline in RP. Although the sample size was small and must be interpreted with caution, this reflects the rarity of the RP subgroup in HD and highlights the importance of continuing to study this vulnerable population. Previous studies compared HD progression to HC, but in this exploratory study, we focused on the RP group, expected to perform similarly to HC. Earlier research suggests that individuals with RP may have a very late onset or even remain asymptomatic, with a 40% chance of being symptom-free by age 65, and 30% by age 75 (McDonnell et al., 2021; Quarrell et al., 2007). However, our findings of this small RP group showed decline despite a mean age of 52. Our study underscores the need to focus on the RP population, who—contrary to expectations of resembling HC and despite not meeting clinical criteria for HD—showed deterioration in visual cognition and increased suicidal ideation at the one-year follow-up.

Collectively, our findings reinforce the concept that HD is a multisystemic disorder whose progression begins well before motor onset. The identification of early decline in visual cognition and worsening of neuropsychiatric symptoms—even in asymptomatic individuals—supports their inclusion as key targets for early detection and intervention. These markers could serve as anchors for future staging models, clinical monitoring protocols, and the development of personalized non-pharmacological interventions aimed at maintaining function and QoL in prodromal or at-risk individuals.

### 4.2 Limitations

Concerning limitations, although HD affects males and females equally, our sample had a slight female predominance (56%), and pre-manifest patients were younger. These variables were controlled in the analysis. While our sample size was large for a rare disease, future studies should include more men, RP participants, and those with 27–35 CAG repeats, considered normal but possibly expanding in future generations. Given the small size of the RP group, RP-related findings are exploratory and should not be generalized beyond this sample; larger RP cohorts are needed to confirm these patterns. Subsequent studies could also expand the protocol to include social cognition and further exploring the pathophysiology of apathy, a common yet poorly understood symptom in HD. Considering its complexity and its unique pattern of development in neurodegenerative diseases, further studies are required to explore the neuropathology and pathophysiology in HD to enable development of effective treatments. Additionally, although we observed an expected degree of sample attrition at 1-year follow-up, we cannot fully exclude the possibility that participants lost to follow-up may have differed in unmeasured ways from those who completed the study, which could introduce potential bias. Nonetheless, the use of mixed-effect models mitigates this issue by allowing valid inferences despite missing data. Lastly, although psychiatric symptoms such as depression, apathy, and suicidal ideation were included as part of the HD phenotype and actively assessed, we cannot fully exclude the possibility that severe active episodes may have influenced cognitive performance in some participants.

### 4.3 Conclusion

In conclusion, this study provides a comprehensive longitudinal characterization of visual cognition and neuropsychiatric progression across the HD spectrum, including pre-manifest RP individuals. Our findings clarify differences in motor, neuropsychological, neuropsychiatric, and general health functioning across preclinical and clinical phases over a one-year follow-up. While previous studies have largely focused on general cognition, this work highlights visual cognition, especially visual memory and visual attention, as a promising early marker of disease progression and brings new attention to the early emergence of neuropsychiatric symptoms, particularly suicidal ideation, even in asymptomatic carriers. Understanding how these symptoms evolve and interact with motor and cognitive changes from the earliest stages, including in RP carriers, can inform more timely detection, prevention, and targeted intervention strategies in clinical practice, ultimately aiming to improve QoL across the HD continuum.

## Data Availability

The raw data supporting the conclusions of this article will be made available by the authors upon reasonable request.

## Supplementary material

Supplementary Figure 1 Confirmatory factor analysis figure for neuropsychological domains. BJLO, Benton Judgment of Line Orientation; BVMT-R, Brief Visuospatial Memory Test-revised; GPT, Grooved Pegboard test; M-WCST, Modified Wisconsin Card Sorting Test; SDMT, Symbol Digit Modalities Test; SPCT, Processing speed with the Salthouse Perceptual Comparison Test; Stroop-C, Stroop Colour; Stroop-W, Stroop Word; Stroop-WC, Stroop Word-Colour; TCF, Taylor Complex Figure; TMT-A, Trail Making Test part A; TMT-B, Trail Making Test part B; VOSP, Visual perception with Visual Object and Space Perception Battery.
