# Supplementary figures and images for: Progression of visual cognition and neuropsychiatric symptoms in Huntington’s disease: a 1-year follow-up study across preclinical and clinical phases
Source: Front Psychol. 2025 Oct 6;16:1609403. doi: 10.3389/fpsyg.2025.1609403 (PMC12535969; doi:10.3389/fpsyg.2025.1609403)

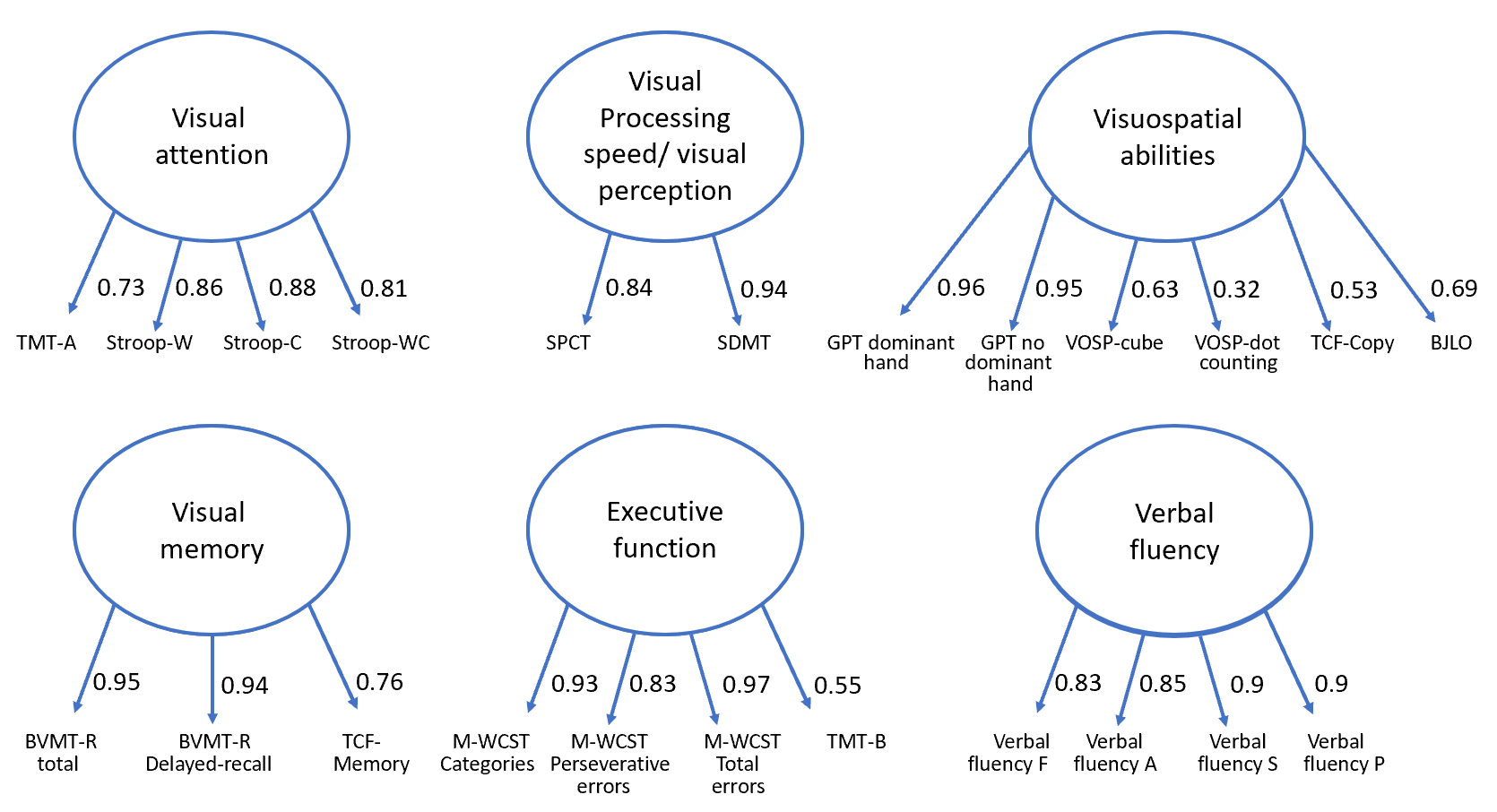

Supplement: Supplementary Figure 1 — Confirmatory factor analysis figure for neuropsychological domains. BJLO, Benton Judgment of Line Orientation; BVMT-R, Brief Visuospatial Memory Test-revised; GPT, Grooved Pegboard test; M-WCST, Modified Wisconsin Card Sorting Test; SDMT, Symbol Digit Modalities Test; SPCT, Processing speed with the Salthouse Perceptual Comparison Test; Stroop-C, Stroop Colour; Stroop-W, Stroop Word; Stroop-WC, Stroop Word-Colour; TCF, Taylor Complex Figure; TMT-A, Trail Making Test part A; TMT-B, Trail Making Test part B; VOSP, Visual perception with Visual Object and Space Perception Battery. [file Image_1.tiff]
