# Supplementary material for: Progression of visual cognition and neuropsychiatric symptoms in Huntington’s disease: a 1-year follow-up study across preclinical and clinical phases
Source: Front Psychol. 2025 Oct 6;16:1609403. doi: 10.3389/fpsyg.2025.1609403 (PMC12535969; doi:10.3389/fpsyg.2025.1609403)
Supplement: Supplementary file 2 [file Table_1.docx]

**Supplementary Table.**

**Supplementary** Table 1. Assessment protocol

| **Instrument** | **Cognitive function** |
| --- | --- |
| **Motor scales** |  |
| Unified Huntingtons’ Disease Rating Scale (UHDRS)-motor | Motor status |
| **Neuropsychological scales** |  |
| Montreal Cognitive Assessment (MoCA) | General cognitive status. Screening for mild cognitive impairment and dementia |
| Word Accentuation Test (WAT) | Premorbid intelligence |
| Taylor Complex Figure (TCF) copy and memory at 3 mins | Visuoconstructive ability and visual memory |
| F-A-S Test | Phonemic Verbal fluency. assesses lexical retrieval and cognitive flexibility. |
| Stroop Test | Selective visual attention. The resistance to interference assesses executive function, including cognitive flexibility, inhibition, and processing speed. |
| Salthouse Perceptual Comparison Test (SPCT) | Visuoperceptive speed and perceptual processing |
| Brief Visuospatial Memory Test-Revised (BVMT-R) | Visuospatial Memory. Visual episodic memory (learning, recall, recognition) |
| Modified Wisconsin Card Sorting Test (M-WCST) | Executive functions and mental flexibility |
| Trail Making Test (TMT) part A and B | Visual attention and executive functions |
| Grooved Pegboard Test (GPT) | Manipulative dexterity, hand dominance, and visuomotor ability |
| Symbol-Digit Modalities Test (SDMT) | Visual attention and visual processing speed |
| Benton Judgment of Line Orientation Test (BJLO) | Visuospatial ability |
| Subtest of the Visual Object and Space Perception Battery (VOSP): Point Counting and Cube Analysis | Visuospatial, visuoperceptive, visuoconstructive abilities. |
| **Neuropsychiatric scales** |  |
| Hospital Anxiety and Depression Scale (HADS) | Anxiety and depression |
| Irritability scale (Snaith) | Irritability |
| Lille apathy rating scale (LARS) | Apathy |
| Columbia Suicide Severity Rating Scale (C-SSRS) | Ideation and Suicide risk |
| **General health** |  |
| Patient Health Questionnaire (PHQ-9) | General health |
| Scale of Quality of life (GENCAT) | Quality of life |
| Instrumental Activities of daily living (IADL) | Activities of daily living |

**Supplementary Table 2. Linear mixed model.**

| **Motor status** | | **Beta** | | **95% CI**^1^ | **p-value** | | | | |
| --- | --- | --- | --- | --- | --- | --- | --- | --- | --- |
| Group | |  | |  |  | | | | |
| Healthy control | | — | | — |  | | | | |
| Pre-manifest | | -0.05 | | -0.25, 0.14 | 0.6 | | | | |
| Early manifest | | -1.2 | | -1.4, -1.0 | <0.001 | | | | |
| Manifest | | -1.8 | | -2.1, -1.6 | <0.001 | | | | |
| Reduced penetrance | | -0.02 | | -0.45, 0.40 | >0.9 | | | | |
| Visit | |  | |  |  | | | | |
| 1 | | — | | — |  | | | | |
| 2 | | -0.07 | | -0.15, 0.02 | 0.14 | | | | |
| Premorbid intelligence | | 0.02 | | 0.01, 0.04 | 0.004 | | | | |
| Daily life activities baseline | | 0.15 | | 0.09, 0.22 | <0.001 | | | | |
| Suicide ideation baseline | | 0.07 | | 0.01, 0.12 | 0.013 | | | | |
| Group * Visit | |  | |  |  | | | | |
| Pre-manifest * 2 | | -0.02 | | -0.18, 0.13 | 0.8 | | | | |
| early manifest * 2 | | -0.27 | | -0.44, -0.09 | 0.003 | | | | |
| manifest * 2 | | -0.13 | | -0.33, 0.07 | 0.2 | | | | |
| Reduced penetrance * 2 | | -0.04 | | -0.35, 0.28 | 0.8 | | | | |
| **General Cognition** | |  | |  |  | | | | |
| Group | |  | |  |  | | | | |
| Healthy control | | — | | — | — | | | | |
| Pre-manifest | | -0.13 | | -0.39, 0.14 | 0.4 | | | | |
| Early manifest | | -0.73 | | -1.0, -0.43 | <0.001 | | | | |
| Manifest | | -0.78 | | -1.1, -0.44 | <0.001 | | | | |
| Reduced penetrance | | 0.26 | | -0.31, 0.82 | 0.4 | | | | |
| Visit | |  | |  |  | | | | |
| 1 | | — | | — | — | | | | |
| 2 | | 0.00 | | -0.16, 0.17 | >0.9 | | | | |
| Age | | -0.02 | | -0.03, -0.01 | <0.001 | | | | |
| Premorbid intelligence | | 0.07 | | 0.05, 0.09 | <0.001 | | | | |
| Daily life activities baseline | | 0.24 | | 0.15, 0.33 | <0.001 | | | | |
| Group * Visit | |  | |  |  | | | | |
| Pre-manifest * 2 | | 0.07 | | -0.21, 0.35 | 0.6 | | | | |
| Early manifest * 2 | | -0.22 | | -0.52, 0.09 | 0.2 | | | | |
| Manifest * 2 | | -0.04 | | -0.38, 0.31 | 0.8 | | | | |
| Reduced penetrance * 2 | | -0.10 | | -0.67, 0.46 | 0.7 | | | | |
| **Visual attention** | |  |  | | |  | |  |  |
| Group | |  |  | | |  | |  |  |
| Healthy control | | — | — | | |  | |  |  |
| Pre-manifest | | -0.06 | -0.26, 0.14 | | | 0.5 | |  |  |
| Early manifest | | -0.43 | -0.64, -0.21 | | | <0.001 | |  |  |
| Manifest | | -0.41 | -0.65, -0.16 | | | 0.001 | |  |  |
| Reduced penetrance | | -0.07 | -0.49, 0.34 | | | 0.7 | |  |  |
| Visit | |  |  | | |  | |  |  |
| 1 | | — | — | | | — | |  |  |
| 2 | | 0.19 | 0.06, 0.32 | | | 0.004 | |  |  |
| Age | | -0.02 | -0.02, -0.01 | | | <0.001 | |  |  |
| Premorbid intelligence | | 0.04 | 0.02, 0.05 | | | <0.001 | |  |  |
| Apathy invert baseline | | 0.08 | 0.02, 0.13 | | | 0.011 | |  |  |
| Group * Visit | |  |  | | |  | |  |  |
| Pre-manifest * 2 | | -0.02 | -0.24, 0.19 | | | 0.8 | |  |  |
| Early manifest * 2 | | -0.55 | -0.80, -0.31 | | | <0.001 | |  |  |
| Manifest * 2 | | -0.96 | -1.2, -0.69 | | | <0.001 | |  |  |
| Reduced penetrance * 2 | | -0.14 | -0.57, 0.29 | | | 0.5 | |  |  |
|  | |  |  | | |  | |  |  |
| **Visual processing speed/visual perception** | | | | | | |  | |  |
| Group | | |  |  | | |  | |  |
| Healthy control | | | — | — | | |  | |  |
| Pre-manifest | | | -0.10 | -0.32, 0.12 | | | 0.4 | |  |
| Early manifest | | | -0.85 | -1.1, -0.61 | | | <0.001 | |  |
| Manifest | | | -1.1 | -1.3, -0.80 | | | <0.001 | |  |
| Reduced penetrance | | | -0.18 | -0.64, 0.28 | | | 0.5 | |  |
| Visit | | |  |  | | |  | |  |
| 1 | | | — | — | | |  | |  |
| 2 | | | 0.03 | -0.07, 0.14 | | | 0.5 | |  |
| Age | | | -0.03 | -0.04, -0.02 | | | <0.001 | |  |
| Years of education | | | 0.03 | 0.00, 0.05 | | | 0.022 | |  |
| Premorbid intelligence | | | 0.06 | 0.04, 0.08 | | | <0.001 | |  |
| Group * Visit | | |  |  | | |  | |  |
| Pre-manifest * 2 | | | -0.07 | -0.23, 0.10 | | | 0.4 | |  |
| Early manifest * 2 | | | -0.12 | -0.31, 0.07 | | | 0.2 | |  |
| Manifest * 2 | | | -0.16 | -0.38, 0.06 | | | 0.2 | |  |
| Reduced penetrance * 2 | | | -0.14 | -0.48, 0.20 | | | 0.4 | |  |
| **Visuospatial abilities** | |  |  | | |  | |  |  |
| Group | |  |  | | |  | |  |  |
| Healthy control | | — | — | | |  | |  |  |
| Pre-manifest | | -0.09 | -0.26, 0.08 | | | 0.3 | |  |  |
| Early manifest | | -0.74 | -0.92, -0.56 | | | <0.001 | |  |  |
| Manifest | | -1.2 | -1.4, -0.94 | | | <0.001 | |  |  |
| Reduced penetrance | | -0.10 | -0.45, 0.24 | | | 0.6 | |  |  |
| Visit | |  |  | | |  | |  |  |
| 1 | | — | — | | |  | |  |  |
| 2 | | -0.09 | -0.18, -0.01 | | | 0.027 | |  |  |
| Sex | |  |  | | |  | |  |  |
| Women | | — | — | | |  | |  |  |
| Men | | 0.15 | 0.03, 0.27 | | | 0.014 | |  |  |
| Age | | -0.01 | -0.02, -0.01 | | | <0.001 | |  |  |
| Premorbid intelligence | | 0.04 | 0.03, 0.06 | | | <0.001 | |  |  |
| QoL baseline | | -0.05 | -0.10, 0.00 | | | 0.034 | |  |  |
| Apathy baseline | | 0.09 | 0.04, 0.14 | | | <0.001 | |  |  |
| Group * Visit | |  |  | | |  | |  |  |
| Pre-manifest * 2 | | 0.04 | -0.10, 0.18 | | | 0.6 | |  |  |
| Early manifest * 2 | | 0.23 | 0.08, 0.39 | | | 0.004 | |  |  |
| Manifest * 2 | | 0.13 | -0.05, 0.31 | | | 0.15 | |  |  |
| Reduced penetrance * 2 | | 0.16 | -0.13, 0.46 | | | 0.3 | |  |  |
| **Visual memory** | | **Beta** | **95% CI**^1^ | | | **p-value** | |  |  |
| Group | |  |  | | |  | |  |  |
| Healthy control | | — | — | | |  | |  |  |
| Pre-manifest | | -0.26 | -0.50, -0.02 | | | 0.033 | |  |  |
| Early anifest | | -1.0 | -1.3, -0.77 | | | <0.001 | |  |  |
| Manifest | | -1.1 | -1.4, -0.76 | | | <0.001 | |  |  |
| Reduced penetrance | | 0.49 | -0.01, 1.0 | | | 0.056 | |  |  |
| Visit | |  |  | | |  | |  |  |
| 1 | | — | — | | |  | |  |  |
| 2 | | -0.09 | -0.24, 0.06 | | | 0.3 | |  |  |
| Age | | -0.02 | -0.03, -0.02 | | | <0.001 | |  |  |
| Premorbid intelligence | | 0.05 | 0.03, 0.07 | | | <0.001 | |  |  |
| QoL baseline | | 0.12 | 0.06, 0.19 | | | <0.001 | |  |  |
| Irritability baseline | | -0.06 | -0.13, 0.01 | | | 0.10 | |  |  |
| Group * Visit | |  |  | | |  | |  |  |
| Pre-manifest * 2 | | -0.16 | -0.42, 0.10 | | | 0.2 | |  |  |
| Early manifest * 2 | | 0.41 | 0.13, 0.70 | | | 0.005 | |  |  |
| Manifest * 2 | | 0.32 | 0.01, 0.64 | | | 0.045 | |  |  |
| Reduced penetrance * 2 | | -0.46 | -0.97, 0.05 | | | 0.074 | |  |  |
| **Executive functions** | | **Beta** | | **95% CI**^1^ | **p-value** | | | | |
| Group | |  | |  |  | | | | |
| Healthy control | | — | | — |  | | | | |
| Pre-manifest | | -0.24 | | -0.51, 0.04 | 0.10 | | | | |
| Early manifest | | -0.65 | | -0.95, -0.35 | <0.001 | | | | |
| Manifest | | -1.3 | | -1.6, -0.94 | <0.001 | | | | |
| Reduced penetrance | | 0.04 | | -0.48, 0.56 | 0.9 | | | | |
| Visit | |  | |  |  | | | | |
| 1 | | — | | — |  | | | | |
| 2 | | -0.02 | | -0.03, -0.02 | <0.001 | | | | |
| Age | | -0.02 | | -0.03, -0.01 | <0.001 | | | | |
| Years of education | | 0.05 | | 0.02, 0.08 | 0.003 | | | | |
| QoL baseline | | 0.00 | | 0.00, 0.00 | 0.13 | | | | |
| Daily life activities baseline | | 0.00 | | 0.00, 0.00 | 0.3 | | | | |
| Group * Visit | |  | |  |  | | | | |
| Pre-manifest * 2 | | 0.00 | | -0.01, 0.00 | 0.4 | | | | |
| Early manifest * 2 | | -0.01 | | -0.02, -0.01 | <0.001 | | | | |
| Manifest * 2 | | -0.02 | | -0.03, -0.01 | <0.001 | | | | |
| Reduced penetrance * 2 | | 0.00 | | -0.01, 0.01 | 0.9 | | | | |
| **Verbal fluency** | | | **Beta** | **95% CI**^1^ | | **p-value** | |  |  |
| Group | | |  |  | |  | |  |  |
| Healthy control | | | — | — | |  | |  |  |
| Pre-manifest | | | -0.12 | -0.37, 0.13 | | 0.4 | |  |  |
| Early manifest | | | -0.71 | -0.99, -0.43 | | <0.001 | |  |  |
| Manifest | | | -0.76 | -1.1, -0.45 | | <0.001 | |  |  |
| Reduced penetrance | | | 0.34 | -0.20, 0.88 | | 0.2 | |  |  |
| Visit | | |  |  | |  | |  |  |
| 1 | | | — | — | |  | |  |  |
| 2 | | | -0.07 | -0.18, 0.04 | | 0.2 | |  |  |
| Years of education | | | 0.03 | 0.01, 0.06 | | 0.014 | |  |  |
| Premorbid intelligence | | | 0.07 | 0.05, 0.09 | | <0.001 | |  |  |
| Apathy baseline | | | 0.07 | 0.01, 0.12 | | 0.019 | |  |  |
| Group * Visit | | |  |  | |  | |  |  |
| Pre-manifest * 2 | | | 0.18 | 0.00, 0.36 | | 0.051 | |  |  |
| Early manifest * 2 | | | -0.16 | -0.36, 0.04 | | 0.12 | |  |  |
| Manifest * 2 | | | -0.12 | -0.35, 0.10 | | 0.3 | |  |  |
| Reduced penetrance * 2 | | | -0.05 | -0.42, 0.31 | | 0.8 | |  |  |
| **Anxiety and depression** | | |  |  | | |  | |  |
| Group | | |  |  | | |  | |  |
| Healthy control | | | — | — | | |  | |  |
| Pre-manifest | | | -0.33 | -0.71, 0.05 | | | 0.089 | |  |
| Early manifest | | | -0.33 | -0.74, 0.08 | | | 0.11 | |  |
| Manifest | | | -0.49 | -0.92, -0.07 | | | 0.024 | |  |
| Reduced penetrance | | | -0.07 | -0.87, 0.74 | | | 0.9 | |  |
| Visit | | |  |  | | |  | |  |
| 1 | | | — | — | | |  | |  |
| 2 | | | -0.04 | -0.31, 0.22 | | | 0.7 | |  |
| Sex | | |  |  | | |  | |  |
| Women | | | — | — | | | — | |  |
| Men | | | 0.43 | 0.18, 0.68 | | | <0.001 | |  |
| Age | | | -0.01 | -0.02, 0.00 | | | 0.016 | |  |
| Group * Visit | | |  |  | | |  | |  |
| Pre-manifest * 2 | | | 0.21 | -0.24, 0.65 | | | 0.4 | |  |
| Early manifest * 2 | | | 0.10 | -0.39, 0.59 | | | 0.7 | |  |
| Manifest * 2 | | | 0.01 | -0.51, 0.54 | | | >0.9 | |  |
| Reduced penetrance * 2 | | | -0.32 | -1.2, 0.57 | | | 0.5 | |  |
| **Irritability** | | |  |  | |  | |  |  |
| Group | | |  |  | |  | |  |  |
| Healthy control | | | — | — | |  | |  |  |
| Pre-manifest | | | -0.17 | -0.55, 0.21 | | 0.4 | |  |  |
| Early manifest | | | 0.04 | -0.38, 0.46 | | 0.8 | |  |  |
| Manifest | | | -0.39 | -0.83, 0.05 | | 0.081 | |  |  |
| Reduced penetrance | | | -0.32 | -1.2, 0.51 | | 0.4 | |  |  |
| Visit | | |  |  | |  | |  |  |
| 1 | | | — | — | |  | |  |  |
| 2 | | | -0.04 | -0.27, 0.19 | | 0.7 | |  |  |
| Sex | | |  |  | |  | |  |  |
| Women | | | — | — | | — | |  |  |
| Men | | | 0.27 | 0.00, 0.54 | | 0.051 | |  |  |
| Years of education | | | 0.04 | 0.00, 0.08 | | 0.032 | |  |  |
| Group * Visit | | |  |  | |  | |  |  |
| Pre-manifest * 2 | | | 0.39 | 0.01, 0.77 | | 0.047 | |  |  |
| Early manifest * 2 | | | -0.18 | -0.60, 0.25 | | 0.4 | |  |  |
| Manifest * 2 | | | -0.04 | -0.50, 0.42 | | 0.9 | |  |  |
| Reduced penetrance * 2 | | | -0.03 | -0.80, 0.74 | | >0.9 | |  |  |
| **Apathy** | | |  |  | | |  | |  |
| Group | | |  |  | | |  | |  |
| Healthy control | | | — | — | | |  | |  |
| Pre-manifest | | | -0.21 | -0.57, 0.14 | | | 0.2 | |  |
| Early manifest | | | -0.59 | -0.98, -0.20 | | | 0.003 | |  |
| Manifest | | | -1.1 | -1.5, -0.71 | | | <0.001 | |  |
| Reduced penetrance | | | 0.06 | -0.70, 0.83 | | | 0.9 | |  |
| Visit | | |  |  | | |  | |  |
| 1 | | | — | — | | |  | |  |
| 2 | | | -0.04 | -0.31, 0.24 | | | 0.8 | |  |
| Years of education | | | 0.03 | 0.00, 0.07 | | | 0.030 | |  |
| Group * Visit | | |  |  | | |  | |  |
| Pre-manifest * 2 | | | -0.10 | -0.55, 0.36 | | | 0.7 | |  |
| Early manifest * 2 | | | 0.20 | -0.31, 0.71 | | | 0.4 | |  |
| Manifest * 2 | | | 0.04 | -0.50, 0.59 | | | 0.9 | |  |
| Reduced penetrance * 2 | | | -0.47 | -1.4, 0.46 | | | 0.3 | |  |
| **Suicide ideation** | | |  |  | |  | |  |  |
| Group | | |  |  | |  | |  |  |
| Healthy control | | | — | — | |  | |  |  |
| Pre-manifest | | | 0.09 | -0.29, 0.46 | | 0.6 | |  |  |
| Early manifest | | | -0.25 | -0.67, 0.16 | | 0.2 | |  |  |
| Manifest | | | -0.29 | -0.72, 0.15 | | 0.2 | |  |  |
| Reduced penetrance | | | -0.65 | -1.5, 0.16 | | 0.12 | |  |  |
| Visit | | |  |  | |  | |  |  |
| 1 | | | — | — | |  | |  |  |
| 2 | | | -0.03 | -0.25, 0.19 | | 0.8 | |  |  |
| Group * Visit | | |  |  | |  | |  |  |
| Pre-manifest * 2 | | | 0.05 | -0.32, 0.42 | | 0.8 | |  |  |
| Early manifest * 2 | | | 0.29 | -0.11, 0.69 | | 0.2 | |  |  |
| Manifest * 2 | | | -0.28 | -0.73, 0.17 | | 0.2 | |  |  |
| Reduced penetrance * 2 | | | -0.33 | -1.1, 0.40 | | 0.4 | |  |  |
| **QoL** | | |  |  | |  | |  |  |
| Group | | |  |  | |  | |  |  |
| Healthy control | | | — | — | |  | |  |  |
| Pre-manifest | | | -0.17 | -0.55, 0.22 | | 0.4 | |  |  |
| Early manifest | | | -0.19 | -0.61, 0.23 | | 0.4 | |  |  |
| Manifest | | | -0.97 | -1.4, -0.53 | | <0.001 | |  |  |
| Reduced penetrance | | | -0.10 | -0.93, 0.74 | | 0.8 | |  |  |
| Visit | | |  |  | |  | |  |  |
| 1 | | | — | — | |  | |  |  |
| 2 | | | -0.09 | -0.36, 0.19 | | 0.5 | |  |  |
| Group * Visit | | |  |  | |  | |  |  |
| Pre-manifest * 2 | | | -0.06 | -0.53, 0.40 | | 0.8 | |  |  |
| Early manifest * 2 | | | -0.03 | -0.54, 0.48 | | >0.9 | |  |  |
| Manifest * 2 | | | 0.56 | 0.01, 1.1 | | 0.047 | |  |  |
| Reduced penetrance * 2 | | | 0.16 | -0.78, 1.1 | | 0.7 | |  |  |
| **Daily life functioning** | | |  |  | |  | |  |  |
| Group | | |  |  | |  | |  |  |
| Healthy control | | | — | — | |  | |  |  |
| Pre-manifest | | | -0.05 | -0.96, 0.85 | | >0.9 | |  |  |
| Early manifest | | | -0.26 | -1.2, 0.66 | | 0.6 | |  |  |
| Manifest | | | -1.6 | -2.5, -0.61 | | 0.002 | |  |  |
| Reduced penetrance | | | -0.06 | -1.3, 1.1 | | >0.9 | |  |  |
| Visit | | |  |  | |  | |  |  |
| 1 | | | — | — | |  | |  |  |
| 2 | | | -0.02 | -0.88, 0.84 | | >0.9 | |  |  |
| Father | | | — | — | |  | |  |  |
| Mother | | | 0.42 | 0.02, 0.81 | | 0.038 | |  |  |
| Premorbid intelligence | | | 0.05 | 0.01, 0.09 | | 0.014 | |  |  |
| Group * Visit | | |  |  | |  | |  |  |
| Pre-manifest * 2 | | | -0.18 | -1.1, 0.74 | | 0.7 | |  |  |
| Early manifest * 2 | | | -0.04 | -0.98, 0.89 | | >0.9 | |  |  |
| Manifest * 2 | | | -0.35 | -1.3, 0.63 | | 0.5 | |  |  |
| Reduced penetrance * 2 | | | -0.01 | -1.2, 1.2 | | >0.9 | |  |  |
|  | | |  |  | |  | |  |  |

Note: CI = Confidence Interval

**Supplementary Table 3. Individual progression of key outcomes in the RP group (baseline to 1-year follow-up).**

| **Domains** | **RP01** | **RP02** | **RPO3** | **RP04** | **RP05** | **RP06** |
| --- | --- | --- | --- | --- | --- | --- |
| Motor status | \| 0.05 \| \| --- \| | 0.16 | 0.00 | -0.58 | 0.00 | -0.16 |
| General cognition | -0.25 | -0.46 | -0.03 | 0.60 | 0.39 | -0.88 |
| Verbal Fluency | -0.18 | -0.82 | 0.39 | -0.45 | 0.17 | -0.08 |
| Executive function | -0.02 | -0.03 | -0.01 | -0.04 | -0.02 | -0.02 |
| Visual memory | -0.17 | -0.76 | -1.11 | 0.24 | -0.65 | -0.78 |
| Visuospatial abilities | -0.01 | 0.11 | 0.09 | 0.10 | 0.03 | 0.04 |
| Visual attention | -0.81 | 0.29 | 0.41 | 0.05 | 0.19 | -0.07 |
| Visual processing speed/ visual perception | -0.23 | -0.35 | 0.46 | -0.08 | -0.39 | -0.03 |
| Anxiety and depression | 0.23 | -0.14 | -0.67 | 2.08 | 0.58 | 0.10 |
| Irritability | -1.41 | 0.07 | -0.26 | 0.48 | 0.23 | 1.28 |
| Apathy | 0.12 | 0.13 | -0.35 | 2.39 | 0.63 | 0.14 |
| Suicide ideation | 0.11 | 0.11 | 0.11 | 0.11 | -0.29 | 2.01 |
| General health | 0.16 | 0.16 | 0.00 | -1.48 | -0.33 | 0.00 |
| QoL | 0.38 | -0.06 | -0.42 | -0.01 | -0.06 | 0.62 |
| Daily life activities | 0.72 | 0.02 | 0.02 | 0.02 | -0.63 | 0.02 |

Note: Δ Z-score = change in standardized score from baseline to 1-year follow-up (Δ Z-score = Follow-up – Baseline; negative values indicate worsening).
